# Mycotoxins and Fungal Toxins: Current Status and Future Perspectives

**DOI:** 10.3390/toxins17040176

**Published:** 2025-04-03

**Authors:** Jianhua Wang, Josefa Tolosa, Wenyu Wang, Xianli Yang

**Affiliations:** 1 Institute for Agro-Food Standards and Testing Technology, Shanghai Academy of Agricultural Sciences, 1000 Jinqi Road, Shanghai 201403, China; m230100125@st.shou.edu.cn; 2 Laboratory of Toxicology, Department of Preventive Medicine and Public Health, Food Sciences, Toxicology and Forensic Medicine, University of Valencia, 46100 Buriassot, Valencia, Spain; josefa.tolosa@uv.es

## 1. Introduction

Many toxigenic fungi are devastating pathogens of crop, fruit, and vegetable diseases worldwide. Serious yield losses in grains and fruits occur annually due to fungal diseases in the field and during storage. In addition to reducing agricultural product yields, devastating fungal diseases can also lead to the contamination of consumption goods with toxic secondary metabolites which pose a threat to human and animal health. Food/feed safety is by far the greatest concern as infected grains are often contaminated with various mycotoxins, such as trichothecenes, aflatoxins, and patulin, and these toxic secondary metabolites can also co-occur. In recent years, the impact of mycotoxins on food safety and human health has aroused considerable public concern. Toxigenic fungi and mycotoxins therefore pose a significant threat to the global food supply and food/feed security.

## 2. There Are Various Types of Mycotoxins Which Are Key Biological Hazards in the Field of Food and Feed Safety

Mycotoxins have attracted global attention because of their wide-ranging appearance in various grains, nuts, fruit juices, and related products. It was estimated by Eskola et al. [1] that approximately 25% of the world’s food/feed commodities are contaminated by mycotoxins annually. On the other hand, multiple toxic effects (emetic, anorexic, immunosuppressive, and even death in cases of severe exposure) induced by these secondary metabolites toward humans and animals have also aroused considerable public concern. Furthermore, several mycotoxins have been shown to behave as virulence factors in plants, allowing fungi to colonize and spread in host tissues.

There is currently a wide range of identified fungal toxins, the most common being aflatoxins, trichothecenes, zearalenone (ZEN), fumonisins, ochratoxin, and penicillin. Members of the *Fusarium* genus represent a large group of toxigenic fungi, which have been reported to produce at least eight kinds of mycotoxins with toxicological significance, including trichothecenes, ZEN, fumonisins, fusarin C, moniliformin, enniatins, beauvericin, and fusaproliferin. Toxigenic fungi in the section of *Aspergillus flavi* synthesize four main molecules, aflatoxin B1, B2, G1, and G2, all of which can yield other derivatives when metabolized by animals after their ingestion [2,3].

Furthermore, the identification of novel fungal toxins in recent years has presented new and significant threats to food and feed safety, such as masked/modified mycotoxins and novel toxins. Modifications of trichothecenes, for example, glycosylation on the C-3 of deoxynivalenol (DON), are more common in nature; this phenomenon has primarily been found in many plants [4]. Most recently, two HT-2-alpha-glucosides produced by *Fusarium sporotrichilides* were identified on a rice medium [5]. Different modified forms of mycotoxins have been found in nature. For example, two kinds of sulfate conjugation of DON, DON-15S and DON-3S, were detected in wheat treated with DON in 2015 [6]. It is worth noting that most mycotoxin modifiers are also easily reduced to the non-modified form in animal intestines or even during food processing [4,6,7,8]. In addition, recently, several novel mycotoxins were identified, for example, NX-2 and NX-3 produced by *Fusarium graminearum* [9,10]. It must be pointed out that among these toxic secondary metabolites, most are currently not regulated in cereals and related food and feed by governments; the toxicity data investigated should provide a robust framework for setting their maximum residue limit values and protecting the health of human beings and animals. There is still a long way to go in the toxicological assessment of different mycotoxins and the development of related limit standards.

## 3. Significant Differences Were Observed in the Species Composition and Genetic Diversity of Toxigenic Fungi and Their Mycotoxins

The biosynthesis of different mycotoxins is complex, being highly influenced by the genetic basis. One fungus has the capability to produce multiple types of mycotoxins in infected matrices. On the other hand, certain toxins can be produced by different pathogenic fungi. For example, the *Fusarium graminearum* clade, *Fusarium culmorum*, and *Fusarium cerealis* are the main sources of DON and ZEN contamination in wheat, maize, and other cereal grains. Meanwhile, fumonisins can be produced by *Fusarium verticillioides* and *Fusarium proliferatum*. Moreover, several subsets of strain-specific trichothecene genotypes have been identified both in the type A and type B trichothecene-producing fusaria. For example, according to the trichothecene production profiles, *Fusarium graminearum* clade strains were subdivided into three different genotypes/chemotypes: the 3ADON genotype/chemotype that can produce DON and 3ADON; the 15ADON genotype/chemotype that can produce DON and 15ADON; and the NIV genotype/chemotype that can produce NIV and 4ANIV [11,12,13,14,15]. *Fusarium graminearum* clade strains typically produce one of three strain-specific profiles of type B trichothecenes, whereas no *Fusarium graminearum* clade strain was reported to produce type A trichothecene prior to 2015 [9,10]. Similarly, in *Fusarium goolgardi* (a type A trichothecene-producing species), two different trichothecene genotypes, DAS and T-2, were identified by Roach et al. in 2015 [16].

Toxigenic fungi are not distributed evenly around the climatic areas of the world, and such ecological differences may contribute to establishing specific regional grain contamination. According to surveys, it is clear that the geographic distributions of mycotoxin-producing fungi are significantly influenced by several elements, such as climatic conditions (temperature, sunshine, humidity, etc.), agricultural practices (soil cultivation, nitrogen fertilization, fungicides, crop rotations, etc.), and even trade [17,18,19]. Due to the different mycotoxins produced by these fungi, the regional differences in these pathogens certainly lead to different risks to crop production and food safety. The *Fusarium graminearum* clade has been intensively studied since its chemotype significantly differs from that of other *Fusarium* species. In America, 15ADON producers are a prevalent population, while 3ADON producers are predominant in China [20,21,22]. A recent report by Senatore et al. [23] indicated that members of the *Fusarium tricinctum* species complex are replacing *F. graminearum* in European countries, for example, Italy.

## 4. The Genetic and Biochemical Approaches and Molecular Mechanisms of Mycotoxin Biosynthesis Are Complex

At the moment, the molecular regulatory mechanisms of the biosynthesis of several fungal toxins are rather evident, but there are still many unanswered questions. Containing the genes required for mycotoxin biosynthesis in the genome of a certain strain is the basic genetic basis for its ability to produce specific toxins. It is clear that most secondary metabolite genes are located in a cluster. In *Fusarium* spp., the core genes responsible for trichothecene biosynthesis are located in an approximately 25 kb cluster on chromosome 2 [24]. This cluster includes 15 genes, and its activation is mainly regulated by *Tri6* and *Tri10* [25]. As recently reviewed by our group, the evolutionary process of the core trichothecene biosynthesis gene cluster and specific *Tri* genes is complex in fusaria [26]. As an example, the results of Roach et al. [16] indicated that a single-nucleotide mutation/deletion occurred in the *Tri1* and *Tri16* genes, which gave rise to the differentiation of the DAS and T-2 genotypes in *Fusarium goolgardi*. Thus, all these apparent genetic differences among mycotoxin-producing fungi highlight the need for monitoring and more phenotypic characterizations of *Fusarium* species. Understanding the evolutionary pathways and molecular mechanisms of *Fusarium* and its mycotoxins will benefit the toxic potential and prediction and identification of unknown mycotoxins.

The biosynthesis of mycotoxins is significantly influenced both by the genetic basis and external environmental conditions. Plenty of genes involved in mycotoxin biosynthesis have been identified and functionally characterized in different fungi, especially in *Aspergillus* spp. and *Fusarium* spp. Among *Fusarium* spp., for example, more than 252 genes have been identified to be involved in DON biosynthesis in *Fusarium graminearum*, as of 2019 [12]. The synthesis of mycotoxins, as secondary metabolites, has been proved to be influenced by other complex mechanisms triggered in response to environmental stimuli, including pH, light, nutrient sources, and other stresses, which may activate different cell signaling pathways resulting in the modulation of the expression of genes involved in toxin production [27,28,29,30,31]. Revealing the connection between gene clusters and environmental stimuli in depth may help to define efficient strategies to decrease mycotoxin production.

Hence, this Special Issue, titled “Mycotoxins and Fungal Toxins: Current Status and Future Perspectives” (https://www.mdpi.com/journal/toxins/special_issues/3NDGM73EAY, accessed on 5 March 2025), was formulated to compile current research and future perspectives on all aspects of toxigenic fungi and mycotoxins. The topic was created on 12 August 2021 with the support of the MDPI management team and officially closed on 30 November 2023. In this Special Issue, a total of eleven articles were published by a diverse group of scientists from different countries. Experts from the fields of mycotoxin toxicology, mycotoxin detection and prevention, and mycotoxin biosynthesis molecular mechanisms contributed their newest findings in the form of research articles or reviews. These findings collectively contribute to the ongoing efforts concerning mycotoxins and offer novel insights into future research work.
